# Knowledge, attitudes, and perceptions of a group of Egyptian dental students toward artificial intelligence: a cross-sectional study

**DOI:** 10.1186/s12903-024-05282-7

**Published:** 2025-01-03

**Authors:** Marwa Elchaghaby, Reem Wahby

**Affiliations:** Pediatric Dentistry and Dental Public Health, Faculty of Dentistry, Cairo University, Cairo, Egypt

**Keywords:** Artificial intelligence, Perceptions, Dental students, Knowledge, AI applications, Dentistry

## Abstract

**Introduction:**

Artificial intelligence (AI) applications have increased dramatically across a wide range of domains. Dental students will undoubtedly be impacted by the emergence of AI in dentistry.

**Aim:**

This study aimed to evaluate the knowledge, attitudes, and perceptions of a group of Egyptian dental students toward artificial intelligence.

**Materials and method:**

An online survey was conducted using a questionnaire sent to dental students via Google Forms. The questionnaire comprised 18 questions on participant’s knowledge and perceptions regarding the future of AI in dentistry. The collected data was statistically analyzed.

**Results:**

A total of 384 students answered the questionnaire. Of the participants, (49%) had a basic knowledge of the principles of AI, and (48%) participants were aware of AI usage in dentistry. Social media was the most common information source for AI applications. Most of the participants agreed on the leading role of AI in the advancement of dentistry and disagreed on the ability of AI to replace dentists in the future, (53%) and (44%) respectively. Moreover, (49%) and (52%) respectively of students approved the incorporation of AI applications in undergraduate and postgraduate dental training.

**Conclusion:**

Egyptian dental students are acquainted with AI and its possible applications in dentistry. They consider the use of AI diagnosis exciting and approve of its definitive role in disease prediction. There is a necessity to include, enhance, and increase AI training in dental schools.

**Trial registration:**

This study has been registered in clinical trials. gov with an identifier: NCT06348758.

## Introduction

Artificial intelligence (AI) has gotten much attention in recent years and has developed into an essential component in both modern medicine and society. Artificial intelligence has sifted from being an absolute statistical instrument to being a key driver of modern medicine [1].

The expression "Artificial Intelligence" was first used in 1956 at a conference, although the theory behind it dates back to 1943. The objective was to develop computers that could perform activities that humans could perform [2].

Using algorithms included in decision support systems, artificial intelligence and its subsets, machine learning, and deep learning, have been extended to analyze complex data collected from multiple sources and have been applied to a variety of dental procedures [3, 4].

AI applications in dentistry were first studied in 1992 [5]. While improvements in AI may not seem to have much of an impact on dentistry overall, several fields have greatly benefited from them, like image-based automatic disease identification and other specialized diagnostic systems, as well as the resolution enhancement of images connected to dentistry [6].

AI has been used in dentistry not only to anticipate and prevent dental problems but also to provide value-based care. AI plays a crucial role in dentistry because it has an enormous ability to find and diagnose oral lesions that are not visible to the human eye. Developments in artificial intelligence may lessen the frequency of avoidable surgical procedures in the medical field, enhance quality of life, relieve postoperative problems, and enhance decision-making [7].

The frequency with which AI applications are being employed in clinical care, research, and education is reflected in the current surge in interest in training AI to medical students. Numerous associations have proposed that AI, data sourcing and safety, ethics, and the critical assessment and interpretation of AI applications in healthcare be taught to healthcare workers [8].

Concerning this continuing AI revolution, it is fundamental to make sure that existing and future clinical workers are up to date regarding the ability of this new technology [5].

It is becoming more apparent that dental students have to learn about AI technologies. Consequently, it would be required to conduct a survey among dental undergraduate students in order to assess their opinions and thoughts about how AI might affect the area of dentistry [9].

A number of studies have been done to evaluate dentistry students' knowledge of artificial intelligence (AI), including ones in Brazil [5], India [10], Morocco [11], Peru [12], Saudi Arabia [13, 14], and South Korea [15] Turkey [16] and United Arab Emirates [17].

Amiri et al., (2024) [18] conducted a systematic review and meta-analysis that provided data regarding the attitudes, knowledge, and skills of medical, dental, and nursing students concerning artificial intelligence, evaluating 5789 participants from 24 studies. Overall, 44% of students showed a medium to high level of knowledge of AI applications and principles. Most students, however, knew very little about artificial intelligence. Although students demonstrated moderate knowledge, they generally had positive attitudes towards AI, as 65% of all students agreed with the use of AI in medicine and had a favorable view.

To the best of the authors’ knowledge, no research has been done about Egyptian dental students’ thoughts and attitudes regarding the employment of AI in the dental field. Therefore, this study aimed to evaluate Egyptian dental students' knowledge, attitudes, and perceptions toward AI.

## Methods

### Study design

The present study is a cross-sectional questionnaire study conducted among a group of dental students in Egypt. Using Google Forms, an electronic link to the questionnaire was created and dispersed via email to the participants. The reporting of this observational study was ensured by applying the STROBE principles.

### Ethical aspects

The Helsinki Declaration was followed in the conduct of the current study. The Cairo University Faculty of Dentistry's Ethics Committee of Scientific Research granted ethical permission on 28/11/2023, with approval number (321,123). Students were informed about the purpose of the study and informed consent was requested at the start of participation to answer the online survey questionnaire while maintaining their anonymity and confidentiality according to the survey's settings.

### Sample size

Based on the findings of (Yüzbaşıoğlu, 2020) [16] where dental students responded with a frequency of (48.4%) to the knowledge about AI, the expected sample size (n) was a total of (384) cases by implementing a (95%) confidence interval. Epi info application was employed for sample size calculation.

### Participants

#### Inclusion criteria

- Undergraduate dental students
- Egyptian dental students
- Both genders were included

#### Exclusion criteria

- Students reluctant to participate and refusing to complete the informed consent.
- Students with skipping records, who didn’t respond to all the survey questions.
- 1st and 2nd-year students.
- Graduates students

### Outcomes

This survey aimed to assess Egyptian dental students' knowledge, attitudes, and perceptions towards AI.

### Data sources and measurement

The data collection tool was a pre-prepared- validated English questionnaire based on Yüzbaşıoğlu, 2020 [16] previous work that was pretested on a group of dental students and showed internal consistency of the survey using the Cronbach’s (= 0.83). Yüzbaşıoğlu, 2020 [16] questionnaire was used without modification.

The electronic survey constructed using Google Forms was distributed to dental students fulfilling the eligibility criteria. Two universities were included in this study: the Faculty of Dentistry, Cairo University, and the Egyptian Russian University.

A consent section explaining the study's purpose, survey nature, and voluntary contribution prefaced the questionnaire. Before proceeding with responding to the questionnaire. Informed consent was taken from each participant by answering a (Yes/No) question.

The questionnaire comprised 3 sections and 22 questions. The initial part of the survey gathered data (4 questions) on sociodemographic characteristics such as age, gender, attending University, and dental education. In the subsequent part, the participants were inquired 3 questions about having a basic knowledge of AI working principles, awareness of AI usage in dentistry, and the information source of AI applications. In the third section, the participants were asked to identify their agreement level to 15 statements.

### Bias

To reduce selection bias, all dental students at participating dental schools were encouraged to anonymously complete the survey. To reduce information bias, the nature of the study and its objective were explained to each participant in the same way.

### Statistical analysis

Statistical analysis was performed using the Statistical Package for Social Sciences (SPSS) version 22.0 statistical analysis tool. Data normality was tested using the Kolmogorov–Smirnov test. The normally distributed data were compared using the independent samples t-test and one-way analysis of variance. The chi-square test was used to assess the categorical data. The analysis's results were shown as frequency (percent) for categorical data. For quantitative data, the analysis's results were shown as mean ± standard deviation. A significance level of P < 0.05 was chosen.

## Results

A total of 384 dental students took part in this survey, 241(63%) students from Cairo University and 143 (37%) students from Egyptian Russian University. The mean age of dental students was 19 ± 1.5 years old. Of the participants, 160 (42%) were male and 224 (58%) were female. 83 (21%) of the participants were third-year, 99 (26%) were fourth-year (*n* = 134), and 202 (53%) were fifth-year dental students. The first and second-year students were not invited to fill out the questionnaire.

Regarding the basic knowledge questions of the working principles of AI, 187 (49%) of the participants were knowledgeable about AI, while 197 (51%) were not (yes vs. no answer). 186 (48%) students were aware of AI usage in dentistry, whereas 198 (52%) were not (yes vs. no answer). Social media was the most common information source of AI applications (*n* = 290,76%) followed by “Friends, family” and “Lectures in university” (*n* = 37,9% and *n* = 39,10% respectively), and “Newspaper, magazines” was the least common source (*n* = 18,5%).

There was an insignificant statistical difference between knowledge about AI and gender, type of attending university, and the education year with p values (0.924, 0.442, and 0.105), respectively. Also, an insignificant statistical difference was observed between awareness of AI usage in dentistry and gender, type of attending university, and the education year with *p* values (0.978, 0.459, and 0.701), respectively. Table 1.

**Table 1:** Responses to the questionnaire's second section in relation to demographic data

Variable			**knowledge about AI**			**Awareness of** **AI usage in dentistry**		
	Number (N)	Percentage (%)	Yes	No	Chi-square	Yes	No	Chi-square
Gender								
Male	160	42%	76	84	0.924	81	79	0.978
Female	224	58%	111	113		111	113	
Attending University								
Cairo university	241	63%	121	120	0.442	124	117	0.459
Egyptian Russian university	143	37%	66	77		68	75	
Dental education year								
3rd year	83	21%	45	38	0.105	44	39	0.701
4th year	99	26%	54	45		51	48	
5th year	202	53%	88	114		97	105	

Moreover, most of the participants agreed on the leading role of AI in the advancement of dentistry and disagreed on the ability of AI to replace dentists in the future, (53%) and (44%) respectively.

When asked about the role of AI in the diagnosis and prediction of oral diseases, most students found the use of AI diagnosis exciting and agreed with its definitive role in disease prediction (57% and 33%, respectively).

It was extensively agreed that AI could be used for the diagnosis of dental caries, periodontal diseases, soft tissue lesions, and pathologies in the jaws radiographically (58%, 56%, 49%, and 51% agreement respectively) in contrast to (10%, 9%, 19% and 15% disagreement respectively).

Regarding the importance of AI in implantology, (54%) of students found AI useful, opposite to (3%) of the participants. In addition, the majority of participants (58%) think that AI could be useful in diagnosis and treatment planning in dentistry, and only (1%) disagreed.

Whereas a large number of participants (40%) had no idea about the role of AI in forensic dentistry, (44%) agreed on its usefulness in forensic dentistry, and the majority (62%) found AI useful as a “quality control tool” and in the assessment of treatments success.

Finally, (49%) and (52%) respectively of the participants approved the incorporation of AI applications in undergraduate and postgraduate dental training.

**Fig. 1:**
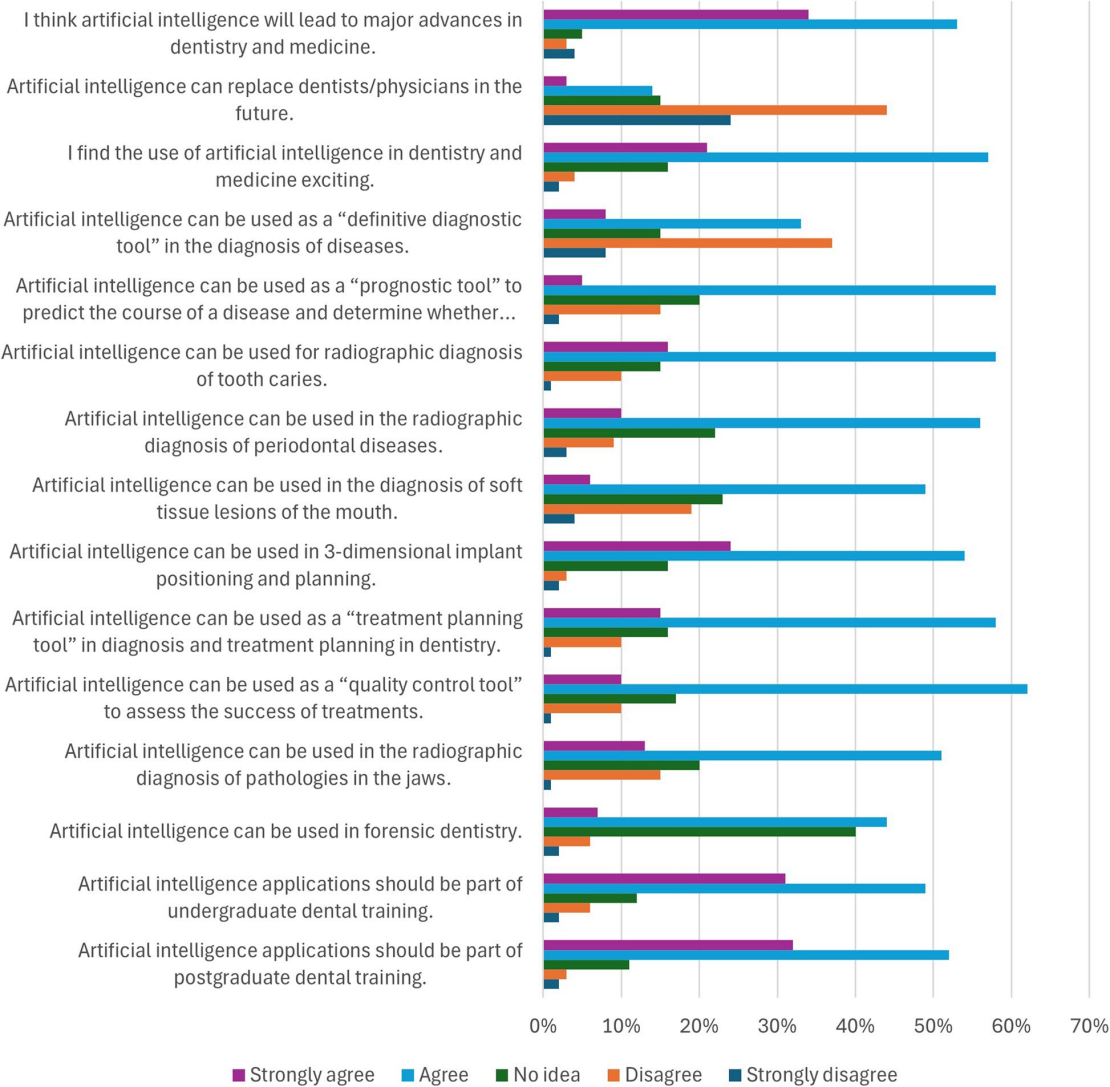
Responses of the questionnaire's third section

Responses to the questionnaire's third section are shown in Fig. 1.

## Discussion

The usage of AI in dentistry is a fast-developing area. Artificial intelligence (AI) applications in practice have challenged the clinical and educational features of dentistry. Nevertheless, AI will unavoidably have a big influence on dental education in the future. The AI paradigm shift makes it clear that universities' dental curricula need to be modified [19].

A survey to ascertain the opinions and attitudes of dentists across all generations is necessary in order to guarantee the proper development and application of dental AI [15].

Several studies in different countries, such as Brazil [5], Turkey [16], Saudi Arabia [13, 14], India [10], Morocco [11], and South Korea [15] have been conducted to assess dental students' knowledge about AI. However, there has been no such attempt in Egypt, and to the authors' knowledge, this is the first survey conducted in Egypt among dental students to collect their knowledge and perceptions of AI.

The male-to-female ratio in the current survey was (42% male compared to 58% female). Similarly, more female participation was reported by Yüzbaşıoğlu, 2020 [16] (59% females and 41% males), Murali et al., 2023 [10] (73% female and 27% male), and Elhijazi and Benyahya, 2023 [11] (71.4% female and 28.9% male). Yet, Khanagar et al., 2021 [13] reported an equal number of participants from both genders (49.2% females and 50.8% males).

In this work, the majority of students were fifth-year dental students (53%), followed by the fourth-year (26%) and third-year (21%). In Yüzbaşıoğlu, 2020 [16] study, second-grade dental students had the majority of participation (36.90%), and fifth-grade dental students had the least participation (8.80%). In Murali et al., 2023 [10] study, the majority of participants (20.7%) were postgraduate students, and the least were third-year students and interns(19.3%). Khanagar et al., 2021 [13] reported that sixth-year dental students and first-year dental students were the most and least to participate in his study (26.7% and 6.9%), respectively.

As recommended by the WHO, health professionals must understand the basic functioning of artificial intelligence to make full and appropriate use of its broad. [11]. In terms of participants' basic understanding of AI's operating principle, (49%) of the participants were knowledgeable about AI, (48%) students were aware of its usage in dentistry.

Close responses were shown by Yüzbaşıoğlu, 2020 [16] as most students were aware of AI; however, the knowledge about its principles was low (48.40%%) and also by Elhijazi and Benyahya, 2023 [11], where 43.6% of students did not know the basic principle of AI. Similarly, a split decision was detected in Khanagar et al., 2021 study [13] (50.1%) with only a one-participant difference. However, higher knowledge levels were reported by Murali et al., 2023 [10] (94.13%) were acquainted with the term artificial intelligence, whereas 73.70% were aware of its basic working concept) and by Kalaimani et al., 2023 [4] (63.5% knew AI and 380 (38%) were aware of its apps. This difference in findings could be attributed to that some dental schools have initiated presenting AI-related programs.

In most of the studies, the participants have learned about AI in social media [Yüzbaşıoğlu, 2020 [16] (76.1%), Khanagar et al., 2021 [13] (40.9%), Aldowah et al., 2024 [14] (78%)]. This could be explained because the majority of people in today's world use social media and almost all have smartphones. Additionally, posts about AI concepts and applications are common on social media [13]. Similarly, in this study, social media was the most prevalent source of AI applications (76%), and “Newspaper, magazines” were the least common source (5%).

When asked if AI will alter the future of dentistry, over half of the participants (53%) agreed on the leading role of AI in the advancement of dentistry. This positive opinion on the utility of dental AI was reported by [Yüzbaşıoğlu, 2020 [16] (85.70%), Khanagar et al., 2021 [13] (72.17%) and Jeong et al., 2024 [15] (64.2%),].

It was reported that one of the most significant industries to use AI is dentistry; nevertheless, understanding of AI is not keeping up with the low usage of AI applications in this field. As a result, some dentists worry that AI could pose a threat to them in the future [20].

In this context, (44%) of students in the current study disagreed on the ability of AI to replace dentists in the future. Similarly, most students in Jeong et al., 2024 [15] did not believe it would replace (71.9%), and half of the participants in Yüzbaşıoğlu, 2020 [16] did not agree that AI could replace themselves soon. However, over half of the students (53.7%) in Elhijazi and Benyahya, 2023 [11] study believed that AI will replace humans in dentistry and expressed moderate to significant concerns about AI. According to Yüzbaşıoğlu, 2020 [16], there are certain difficulties, such as the fact that AI is unable to engage in in-depth conversations with patients in order to win their trust, reassure them, and show empathy. Physicians will still be needed to integrate medical histories, conduct physical examinations, and promote additional discussion in unclear cases.

When questioned about whether the use of AI in dentistry and medicine is exciting, (57%) of students found the use of AI diagnosis exciting. The same opinion was reported by Turkish and Saudi students [13, 16] ( 51% and 47.3%) respectively.

AI systems are starting to provide new insights into healthcare and play a significant role in the diagnosis and prognosis of diseases. These algorithms examine enormous volumes of medical data to find connections and patterns that would be difficult for humans to find [21]. In this term, 33% of the students agreed with AI's definitive role in disease prediction. Also, students in Turkey [16], Saudi Arabia [13], and India [10] agreed that AI can be employed as a prognostic way to predict disease and decide whether there is a recovery chance.

It was generally agreed that AI could be used for the diagnosis of dental caries, periodontal diseases, soft tissue lesions, and pathologies in the jaws radiographically (58%, 56%, 49%, and 51% agreement, respectively). This was in accordance with Turkish [16], Saudi [13], and Indian students [10], who thought that AI could be used for radiographic diagnosis of tooth caries, periodontal diseases, and the diagnosis of soft tissue. In addition, the majority of participants (58%) think that AI could be useful in diagnosis and treatment planning in dentistry. Close responses were reported in Turkey [16] (57.2%), Saudi Arabia (44.2%) [13], and much higher in India [10] (88.69%).

According to the systematic reviews of Khanagar,2021 [22] and Khan, 2024 [23], AI is revolutionary in terms of delivering reliable data for forensic scientific decision-making. AI models can be useful tools for identifying victims of mass disasters and as an additional aid in medico-legal situations while concurrently reducing the time required and the risk of human error. By minimizing the effect of human factors, AI can contribute to more reliable and reproducible outcomes. In this study (44%) of participants agreed on AI's usefulness in forensic dentistry. This was in accordance with studies by Yüzbaşıoğlu, 2020 [16] (52.1%), Khanagar et al., 2021 [13] (38.3%), and Murali et al., 2023 [10] (90.43%).

The wish to integrate and receive more training in AI applied to dentistry in undergraduate and postgraduate dental training was expressed by (49%) and (52%) respectively of participants. One possible explanation for this could be that undergraduate dental students are eager to learn about new dental technology that has the potential to improve treatment outcomes. There was almost universal agreement that AI should be incorporated into the undergraduate and postgraduate curriculums of dental schools.

The majority of the students (96%) in Elhijazi and Benyahya, 2023 [11], (85.43%) in the Murali et al., 2023 [10], and ( 80%) in Kalaimani et al., 2023 [14] studies wished to receive more in-depth training in AI applied to dentistry. Also, (79.80% and 74.60%, respectively) of participants in the Yüzbaşıoğlu, 2020 [16] study granted that subjects about AI should take part in undergraduate and postgraduate dental education. Moreover (59%) of students in Aldowah et al., 2024 [4] and almost half of the participants in Jeong et al., 2024 [15] (49%) studies believed that AI should be included in dental curricula.

Finally, this study is the first attempt to evaluate Egyptian students’ knowledge and perceptions of AI and can serve as a basis for future studies on the subject and for assessing the long-term impact of the integration of artificial intelligence into the educational curricula of dental students. However, the participants were recruited from only two universities, therefore, the chosen participants may not be appropriate representatives of dental students. Future surveys are necessary to monitor shifts in perception over time and give in-depth analytic data, especially considering how quickly artificial intelligence is developing.

## Conclusions

According to the outcomes of this study, a slight majority of dental students were knowledgeable about AI and aware of its usage in dentistry. Students consider the use of AI diagnosis exciting and believe in its pivotal role in disease prediction. Including, enhancing, and increasing AI training in dental schools is necessary.

## Data Availability

All data generated or analyzed during the study are included in this research article.
